# The population genetics of the Jewish people

**DOI:** 10.1007/s00439-012-1235-6

**Published:** 2012-10-10

**Authors:** Harry Ostrer, Karl Skorecki

**Affiliations:** 1 Departments of Pathology and Genetics, Albert Einstein College of Medicine, Bronx, NY 10461 USA; 2 Rappaport Faculty of Medicine and Research Institute, Technion-Israel Institute of Technology, Rambam Health Care Campus, 8 Ha’Aliyah Street, 31096 Haifa, Israel

## Abstract

Adherents to the Jewish faith have resided in numerous geographic locations over the course of three millennia. Progressively more detailed population genetic analysis carried out independently by multiple research groups over the past two decades has revealed a pattern for the population genetic architecture of contemporary Jews descendant from globally dispersed Diaspora communities. This pattern is consistent with a major, but variable component of shared Near East ancestry, together with variable degrees of admixture and introgression from the corresponding host Diaspora populations. By combining analysis of monoallelic markers with recent genome-wide variation analysis of simple tandem repeats, copy number variations, and single-nucleotide polymorphisms at high density, it has been possible to determine the relative contribution of sex-specific migration and introgression to map founder events and to suggest demographic histories corresponding to western and eastern Diaspora migrations, as well as subsequent microevolutionary events. These patterns have been congruous with the inferences of many, but not of all historians using more traditional tools such as archeology, archival records, linguistics, comparative analysis of religious narrative, liturgy and practices. Importantly, the population genetic architecture of Jews helps to explain the observed patterns of health and disease-relevant mutations and phenotypes which continue to be carefully studied and catalogued, and represent an important resource for human medical genetics research. The current review attempts to provide a succinct update of the more recent developments in a historical and human health context.

**Electronic supplementary material:**

The online version of this article (doi:10.1007/s00439-012-1235-6) contains supplementary material, which is available to authorized users.

## Introduction

Since their emergence as a national and religious group in the Middle East over 2,000 years ago (Biran and Naveh 1993), Jews have maintained continuous cultural and religious traditions amid a series of Diasporas (Ben-Sasson 1976). Along the way, others were converted into the Jewish fold. The origins and relatedness of the various Jewish groups have been much speculated upon over the past century. Jews have described themselves as a “people”, based on their shared religion, without a clear indication of the genetic lines of descent since their early history. Albert Einstein captured this uncertainty when he wrote to the Berlin rabbis in 1921 “I notice that the word Jew is ambiguous in that it refers (1) to nationality and origin, (2) to the faith” (Einstein et al. 1987).

With the advent of modern population genetics based on analysis of genetic markers in the second half of the twentieth century, investigators have attempted to categorize the origins and relatedness of Jewish people. Because relatively few polymorphic markers were available at first, the early studies focused on genetic distances between groups and established hierarchies based on these distances (Bonne-Tamir et al. 1978a, b, 1977; Carmelli and Cavalli-Sforza 1979; Karlin et al. 1979; Kobyliansky et al. 1982; Livshits et al. 1991). Population genetics has been enhanced by the identification of millions of polymorphic markers that reside in close proximity to one another along the genome and that vary in their allele frequencies among populations. These discoveries have led to greater precision for estimates of genetic distances. These discoveries have also led to new types of analyses that were not available in the past. The analyses have included deconvolution of ancestry for whole genomes or for segments of individual genomes and analysis of segmental sharing among individuals that provide greater accuracy for estimating their degree of relatedness (Atzmon et al. 2010; Bryc et al. 2010).

At the same time, genetic analyses of diseases have continued in Jewish populations. These have included diseases with a clear Mendelian basis, rare syndromes often identified first in a single family, common conditions that are more prevalent in Jewish populations, and common conditions for which the complexity might be simplified by studying Jewish populations. Interest in studying these disorders has accelerated with the advent of genomic sequence-based personalized medicine research (Ostrer 2011). Here, we provide a description of the population genetics of the Jewish people based on these recent discoveries and a progress report on the genetic basis of diseases.

## History as a guide to understanding Jewish population genetics

The history of the Jewish people from Classical Antiquity onward provides a guide to understanding their population genetics and is most accurately recorded beginning at the time of the Greek and Roman Empires. Up to 6 million Jews are thought to have resided in the Roman Empire, comprising 10 % of the total population (Fishberg 1911). In the period immediately preceding the fall of the Second Temple in 70 CE, adherents to Judaism were located throughout the Roman Empire, to the west, and extended into the Arsacid Empire in the east (Isaac 1998). These Jews are likely to have been the ancestors of the subsequent Jewish Diaspora populations that lived in the Middle East (“Mizrahi”), Europe (“Ashkenazi and Sephardic”) and North Africa (Baron 1952). The number of adherents to Judaism residing outside of the Kingdom of Judea is thought to have greatly exceeded those residing within Judea with the largest communities in Alexandria in Egypt and Antioch in contemporary Turkey. Evidence for these communities remains in the archeological record, such as the well-studied community in Dura-Europos then at the boundary of the Roman Empire and now in present-day Syria (Chi et al. 2011). Most introgression with non-Jews occurred during times of relative liberalism and tolerance, including the Hasmonean period in Classical Antiquity (140–37 BCE) and modern times (Shanks 1988). Introgression between Jewish groups also occurred following the Spanish Inquisition when Sephardic Jews left the Iberian Peninsula (1492–96 CE) and migrated to Italy, the Balkans, Syria, Morocco, and Algeria, often settling within existing Jewish communities (Stillman 1991). Although not sustaining communities that were recognizably Jewish, Sephardic Jews also migrated to the New World and contributed to the formation of contemporary Hispanic and Latino non-Jewish populations (Hordes 2005). Since the fall of the Second Temple and the end of the Judean kingdom in 70 CE, religious law and anti-Semitism in the emerging Christian and Islamic worlds favored marrying within the Jewish fold (Cohen 1999; Goldstein and Evans 2012; Wistrich 2010).

Judaism was also brought outside the Roman Empire to Yemen, Ethiopia, India, and China. Many of these communities were long-standing and were observed by Benjamin of Tudela during his travels of the twelfth century (Benjamin 1983). The origins of these communities have been the subject of considerable speculation. Some communities have been thought to be the descendants of the Lost Tribes that were forced into Assyrian exile following the destruction of the Kingdom of Israel in 622 BCE, although unsupported by historical evidence (Parfitt 2002; Gonen 2002). Some commentators have suggested that these communities may have been established by Jewish traders (usually men) who brought their ideas and genes and converted members of the local population (Goldstein 2008). Within these communities, the contemporary composition may have been influenced by the number and origins of the founders as well as by the subsequent admixture events. Forced and voluntary conversion out of the Jewish faith has been well documented and has left genetic imprint on some contemporary non-Jewish populations (Velez et al. 2011).

## Population genetics as a guide to understanding Jewish history

Early population genetic studies based on blood groups and serum markers provided evidence that most Jewish Diaspora groups originated in the Middle East and that paired Jewish populations were more similar genetically than paired Jewish and non-Jewish populations (Bonne-Tamir et al. 1978a, b, 1977; Carmelli and Cavalli-Sforza 1979; Karlin et al. 1979; Kobyliansky et al. 1982; Livshits et al. 1991). These studies differed in their inferences regarding the degree of admixture with local populations. Subsequent studies of the monoallelic Y chromosomal and mitochondrial DNA haplotypes demonstrated founder effects of both Middle Eastern and local origin, but did not adequately resolve the degree of admixture. To resolve this issue and to improve the understanding about the relatedness of contemporary Jewish groups, our research teams and others have independently performed genome-wide analyses of Diaspora Jewish groups and comparison with neighboring populations (Atzmon et al. 2010; Behar et al. 2010; Campbell et al. 2012; Kopelman et al. 2009; Bray et al. 2010; Listman et al. 2010). These studies varied in the specific populations analyzed and in the number of individuals included from each population. Yet, they came to remarkably similar conclusions, providing evidence for shared genetic ancestries among major Jewish Diaspora groups together with variation in admixture with local populations.

**Fig. 1:**
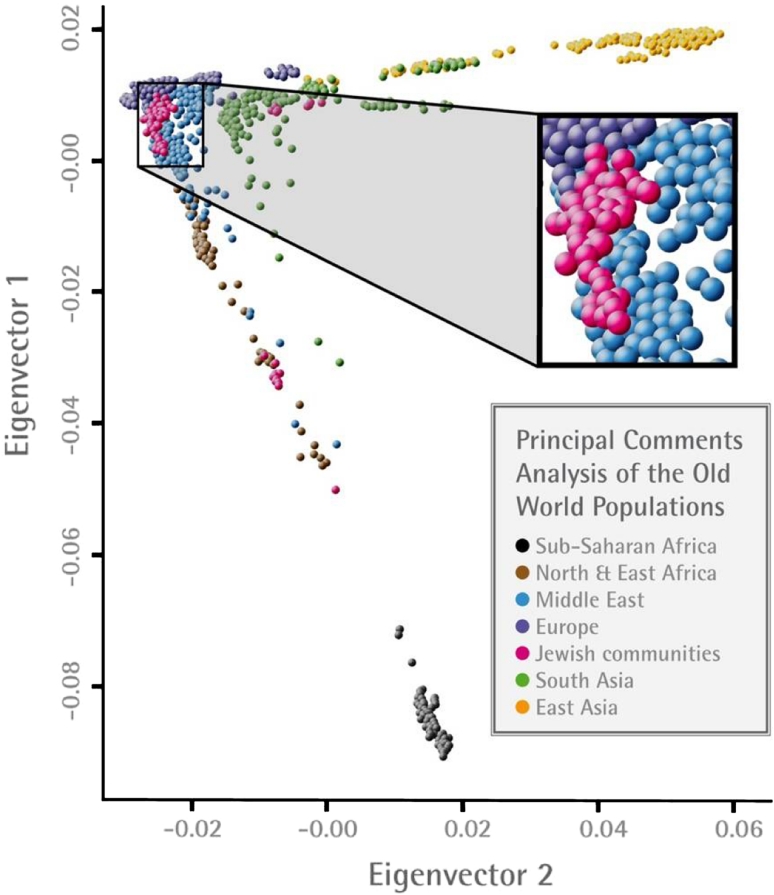
Principal components 1 and 2 analysis of major central cluster of Jewish populations combined with other Old World populations (indicated by *different colored balls*). Figure based on data in Behar et al. (2010), which also provides and illustrates the data for subjects remote from the major central cluster. Blow-up of data for Jewish, European, and Middle Eastern populations is also shown

**Fig. 2:**
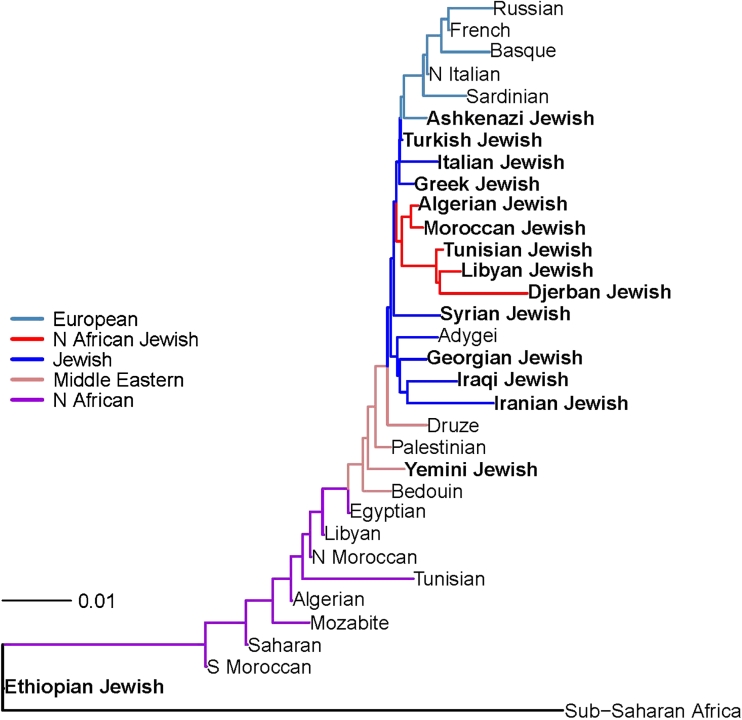
Neighbor-joining tree showing the relationship of European, Jewish, Middle Eastern, and North African populations, using Fst as the distance metric. The neighbor-joining algorithm used Fst as the distance metric input for calculation of a matrix specifying the distance between each pair of groups and then iterates until the tree is resolved and branch lengths discerned. The tree was rooted using the reference mixed Central and Southern African population as an out-group. Major population groups are labeled at the *right*

By principal component analysis, it was observed that the Jewish populations of Europe, North Africa, and the Middle East formed a tight cluster that distinguished them from their non-Jewish neighbors (Fig. 1). Within this central cluster, each of these Jewish populations formed its own subcluster, in addition to the more remote localization of members of some Diaspora communities. The observation of a major central tight cluster was supported by statistical metrics for genetic distances (Fst, allelic sharing distances). Nearest neighbor-joining analysis robustly supported shared origins of most Jewish populations with clearly discernible European/Syrian/North African and Middle Eastern branches (Fig. 2; Campbell et al. 2012). Turkish, Greek, and Italian Jews shared a common branch, with Ashkenazi and Syrian Jews forming connections to this branch. The North African populations added a sub-branch to the European/Syrian branch. In turn, this North African sub-branch bifurcated into Moroccan, Algerian and Tunisian, Djerban, Libyan sub-branches. More detailed PCA analysis showed that the Tunisian Jewish group was identifiable by two clusters, one with proximity to Libyan Jews and the other with proximity to Moroccan Jews. Moreover, by PCA analysis, the North African Jewish populations were orthogonal to contemporary non-Jewish North African populations from Western Sahara, North and South Morocco, Algeria, Tunisia, Libya, and Egypt. The Middle Eastern Jewish branch included the Iranian, Iraqi and Georgian Jews as well as the non-Jewish Adygei.

Even groups that fell outside of the shared Jewish population cluster identified by PCA such as Ethiopian Beta Israel, Yemenite, Indian Bene Israel, and Indian Cochin Jews, formed their own subclusters indicating that they were distinct, homogeneous populations. On the nearest-neighbor-joining tree, the Yemenite Jews were on a branch between Palestinians and Bedouins, and the Ethiopian Beta Israel Jews were on a distinctive distal branch. Uniparental genome region analysis provided additional insights, for example, supporting male predominant Middle East Jewish origins for the Bene Israel population (Behar et al. 2010).

**Fig. 3:**
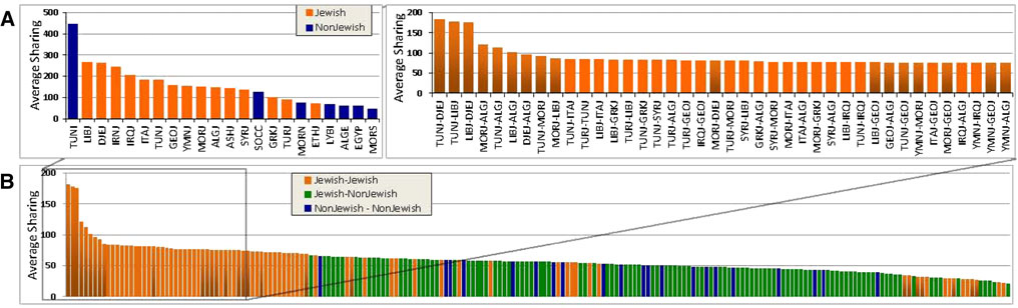
Genome-wide identity-by-descent (IBD) sharing for the average pair of individuals within (**a** *left*) and across populations (**a** *right*, **b**). Genome-wide IBD analysis estimates kinship based on sharing of DNA sequence segment similarities as determined by variable sites across the genome. With the exception of non-Jewish Tunisian samples, IBD sharing is higher within Jewish groups, reflecting higher levels of endogamy. Jewish populations exhibit higher sharing with other Jewish populations than with geographically near groups. The average total sharing across Jewish populations is generally higher than the sharing across other population pairs

The genetic sharing within and among these populations occurred not only at the single-nucleotide polymorphism (SNP) level, but also at the levels of copy number variants (CNVs) and, where studied, identical-by-descent (IBD) segment sharing (Fig. 3; Atzmon et al. 2010; Campbell et al. 2012). The IBD segment sharing was greater within specific Jewish populations and as expected highest among Jewish populations with greater degrees of inbreeding, such as Libyan, Djerban, and Tunisian Jews. In fact, the general degree of sharing within populations was similar to what one might observe for fourth to fifth cousins. This included the Yemenite as well as the Middle Eastern, European and North African Jews. Detailed patterns of segment sharing provided still further insights. Thus, for example, a pattern of more numerous shorter segments shared among Ashkenazi Jews is consistent with a population bottleneck effect (see below). Notably, the degree of sharing between Jewish populations was also greater than the sharing between Jewish and non-Jewish populations. These studies showed that Jews have a tapestry of shared DNA threads with other Jews and that no one thread is sufficient to define Jewish ancestry.

These studies also demonstrated that the history of the Jewish Diasporas could be observed in the genomes of Jewish people by patterns of admixture. A high degree of European admixture (30–60 %) was observed among Ashkenazi, Sephardic, Italian and Syrian Jews. The North African Jewish groups demonstrated North African and Middle Eastern admixture with varying European admixture. The proportion of European admixture among North African Jewish groups increased from east to west, with Moroccan Jews demonstrating the highest proportion. In contrast, the corresponding non-Jewish North African host populations demonstrated substantially higher inferred North African ancestry and less European ancestry.

The closest genetic neighbors to most Jewish groups were the Palestinians, Israeli Bedouins, and Druze in addition to the Southern Europeans, including Cypriots. The genetic clusters formed by each of these non-Jewish Middle Eastern groups reflect their own histories of endogamy. Their proximity to one another and to European and Syrian Jews suggested a shared genetic history of related Semitic and non-Semitic Mediterranean ancestors who followed different religious and tribal affiliations. Earlier studies of Israeli Jewish, Palestinian and Druze populations made a similar observation by demonstrating the proximity of these two non-Jewish populations to Ashkenazi and Iraqi Jews (Rosenberg et al. 2001; Kopelman et al. 2009).

## Monoallelic markers provide insight into founder effects

Monoallelic markers, Y chromosomal and mitochondrial haplogroups, have proven to be very useful for understanding the patrilineal and matrilineal origins of Jewish Diaspora groups. Y chromosomal analysis showed that most Diaspora Jews whose ancestors lived in the Middle East, Europe or North Africa, one to two generations ago, were descended from a smaller group of Middle Eastern men (Hammer et al. 2000). Similar Y chromosomal lineages have been found among Christian and Muslim men who live in the Middle East today. Seven Y chromosome major branches (E3b, G, J1, J2, Q, R1a1, and R1b) that are prevalent among Ashkenazi Jews account for 80 % or more of the total (Behar et al. 2004). Four of these (E3b, G, J1, J2, Q) were part of the ancestral gene pool transmitted by Jews who migrated from the Middle East, whereas R1b and certain sublineages within R1a may have entered the Ashkenazi Jewish population in Europe.

Unique founder events are evident using detailed lineage analyses of these seven Y chromosome biomarkers in the Ashkenazi Jewish population (Behar et al. 2004). The comparative frequencies of these lineages between contemporary Jews to European non-Jews led to an estimate of an overall historical male admixture rate of 5–8 % with European populations, since the founding of the Ashkenazi Jewish population. This is the same as an admixture rate of 0.5 % per generation (Hammer et al. 2000). The presence of European Y chromosomal lineages is the major difference between Ashkenazi Jews and Middle Eastern and Sephardic Jews (Nebel et al. 2001).

Yet this Middle Eastern/European distinction in the Ashkenazi gene pool may be an oversimplification. Some of these Middle Eastern Y chromosomal lineages were brought by Middle Eastern settlers during the Stone and Bronze Ages colonization of Europe, then introduced through admixture between Europeans and Jews (Semino et al. 2000). The most common Ashkenazi Jewish Y chromosomal types of European origin are R1a1 and R1b with frequencies of 7.5 and 10 %, respectively. R1a1 is very common among Russians, Ukrainians, and Sorbs (Slavic speakers in Germany), as well as among certain Central Asian groups. This may be the signal of much-speculated Khazar admixture with Ashkenazi Jews, although the admixture may have occurred with Ukrainians, Poles or Russians (Nebel et al. 2005). However, it should be noted that a Middle Eastern origin for some R1a1 lineages cannot be ruled out. R1b is the most common Y chromosome branch of Atlantic Europe. Its occurrence among Ashkenazi Jews may be an indicator of admixture that occurred in the Rhine Valley prior to the Ashkenazi Jewish migration to Eastern Europe or at later time points in certain locales (Nebel et al. 2005). This branch is also prevalent in Lebanon among the Maronite Christian community and may reflect the admixture with Crusaders following their invasions in the 11–13th centuries CE (Zalloua et al. 2008).

Analysis of Jewish mitochondrial genomes in some Diaspora communities has demonstrated limited genetic diversity and therefore, evidence for strong founder effects. Four mitochondrial haplogroups were found to account for >40 % of the total among Ashkenazi Jews, providing evidence for four founder females, with a limited number of additional female founders accounting for of the remainder (Behar et al. 2008). These haplogroups form the so-called “star phylogenies” around a coalescent, ancestral mitochondrial haplotype. At least some of these founders clearly originate in the Middle East, with an overall pattern similar to that observed for male Ashkenazi Jewish founders. Evidence for female founders has been observed in other Jewish populations. The number of founders and the relative proportion of founders vary greatly from one Jewish Diaspora population to another. There are very few founder lineages among the Jews of Azerbaijan, Georgia, Libya, Mumbai, India, and Belmonte, Portugal, and these lineages account for the majority of mitochondrial haplotypes. In all of these populations, a sole founding mother was sufficient to account for at least 40 % of the contemporary mitochondrial genomes. Among the Jews of Tunisia and Cochin (south India), two founding mothers account for 30 % of the mitochondrial genomes. In the Bulgarian, Turkish, Moroccan, and Ethiopian Jewish communities, there was no evidence for a narrow founder effect. The Bulgarian, Turkish, and Moroccan communities all received large influxes of Jewish refugees following the Spanish Inquisition. The high degree of diversity observed today probably reflects the degree of diversity that was present among the Jews of Spain prior to the expulsion in 1492, and Portugal in 1496. The diversity observed among the Ethiopian Jews reflects the variety of maternal lineages that were present during the founding and propagation of this community in East Africa. By contrast, the Iranian, Iraqi, and Yemenite communities demonstrate a degree of diversity that is intermediate to that observed in the other groups. The communities were all founded at least 2,000 years ago, and the mitochondrial genotypes are not consistent with a narrow founding event with at least six founding mothers in these populations. None of these populations is quite like the Ashkenazi Jews which have a large contemporary population base, but relatively few founders.

With two exceptions, all of the populations had mitochondrial genomes that were of Middle Eastern origin. The Ethiopian mitochondrial genomes were of African origin and the Bene Israel of Indian origin. This demonstrates that Jewish population origins have been determined not only by the flow of genes, but also by the flow of ideas, although this does not exclude the flow of genes from some now undetected founder Jewish women at the time of formation of a new Jewish community. This observation provides some resolution to the queries of the physical anthropologists, Maurice Fishberg and Joseph Jacobs, for why Indian and Ethiopian Jews bear a physical resemblance to their local populations (Fishberg 1911; Jacobs 1899).

## Insights from Jewish disease genetics (please see supplementary table 1)

Genetic analyses of Mendelian diseases were utilized early on as a means of understanding the population genetics of the Jews and subsequently many other genetic isolates. Chaim Sheba and Victor McKusick were key opinion leaders in this regard, as typified by McKusick’s comment, “One of my most cherished memories is of ward rounds with Dr. Chaim Sheba at Tel-Hashomer Hospital in 1964. As we passed from bed to bed, Dr. Sheba would say ‘this is a Moroccan Jew. They are particularly susceptible to diseases A and B’ or ‘this is a Yemenite Jew. They are particularly susceptible to diseases C and D’. It was Dr. Sheba who stimulated my interest in the ethnic distribution of disease” (McKusick 1973). The advent of Israeli statehood and the resulting immigration of Jewish Diaspora groups to Israel provided an unprecedented opportunity for studying these conditions. Richard Goodman described it this way, “After the State of Israel came into being in 1948, waves of new immigrants from over 100 countries throughout the world came by land, sea and air to the country. They were literally met at their ports of entry by teams of physicians and geneticists, seeking to learn about the heritable differences and similarities of these people who had been dispersed for over 2,000 years” (Goodman 1974). These have included X-linked and autosomal dominant and recessive Mendelian disorders that occur in individual families, in specific Jewish Diaspora groups, across two or more Diaspora groups or across Jewish and non-Jewish groups (Goodman 1979; Ostrer 2001). These conditions have been identified, because they have a recognizable phenotype and commonly have an allele frequency exceeding 0.05 in a population of interest. Comprehensive characterization and cataloging has been carried out and is included as part of the Israeli National Genetic Database (http://www.goldenhelix.org/server/israeli/) coordinated by Joel Zlotogora (van Baal et al. 2010). Molecular analysis has usually identified one or two common alleles that account for 70 % or more of the cases. Coalescence theory has demonstrated that these mutations have arisen throughout Jewish history and in some in the pre-Jewish era (Ostrer 2001; Risch et al. 2003). Disease phenotype causing mutations in a given Jewish Diaspora group have been shown to coalesce typically to the founding of that group. Both selection and drift have been proposed as mechanisms for higher frequencies of such mutations within Jewish Diaspora groups. However, the inferred geographic distribution of Ashkenazi Jewish mutation carriers in Europe supported drift over selection (Risch et al. 2003). Likewise, the observation that Ashkenazi Jewish founder mutations did not reject the neutrality hypothesis in the Slatkin–Bertorelle test also supported drift (Slatkin 2004). Malaria resistance for the G6PD^Med^ mutation is the only proven example of a role for selection (Motulsky et al. 1966). However, many of these conditions do seem to cluster into specific categories, including lysosomal storage diseases (Tay-Sachs disease, Niemann-Pick disease, Gaucher disease and mucolipidosis IV), glycogen storage disease (types I and III), clotting factor deficiencies (factor XI deficiency, factor VII deficiency, combined factors V and VIII deficiency), steroid hormone biosynthetic defects (21-hydroxylase deficiency, 11-hydroxylase deficiency, and corticosterone methyloxidase II deficiency), and defects in DNA repair that increase susceptibility to cancer (hereditary breast-ovarian cancer susceptibility caused by mutations in *BRCA1* or *BRCA2*, Bloom syndrome, and Fanconi anemia) (Ostrer 2001). The reason for this clustering of diseases is not known, but does favor the idea of a common selective pressure conferring advantage to the heterozygote state across disease mutations in the same cluster. Contemporary methods of genomic analysis are starting to provide evidence for selection of other variants in the genome without evidence for the selective agents (Bray et al. 2010).

The study of these conditions has been translated into public health genetics initiatives for Jews, and where the same conditions occur, non-Jews alike. These have included genetic screening programs for reproductive risks, presymptomatic identification of disease risk, and treatment of genetic disease. Screening for Tay-Sachs disease has been available for Ashkenazi Jews since the 1970s (Kaback 2001). This has taken many forms, both community-based and medically based. Thousands of people come each year for genetic testing, both pregnant couples trying to decide, whether they should have prenatal diagnosis via amniocentesis and young Orthodox Jewish men and women contemplating marriage, but wanting to obtain genetic information before developing a serious relationship. Over a million people have had Tay-Sachs carrier testing and this has decreased the number of Tay-Sachs births. Currently, 4–5 children are born each year with Tay-Sachs disease, in contrast to the 40–50 annually prior to genetic screening. Ashkenazi Jews are now screened typically for 18 conditions and this number is likely to rise. New genetic testing programs are also being developed for Sephardic and Middle Eastern Jews, with the goal of preempting the tragic conception or birth of a child with a fatal genetic disease. In the past, when a couple had a child with a genetic disease, oftentimes they stopped having children. Screening for genetic diseases has been accepted into the mainstream of Judaism. It should be kept in mind that the growing accessibility of whole-exome and eventually whole genome sequence information is likely to change the landscape of such public initiatives in genetic testing for Jewish and other populations alike.

The second way in which genetic knowledge has impacted medically has been in the treatment of genetic disease. One of the dramatic examples is the treatment of Gaucher disease, a lysosomal storage disease that results in accumulation of glucocerebroside into the lysosomes of cells (Charrow 2009). People with Gaucher disease can be quite disabled with bleeding, bone pain, hip fractures, and large spleens. Knowledge of the genetic defect in Gaucher disease led to a specific therapy in which the enzyme that is missing from the people with the disease is replaced by injection. The results of treatment are dramatic with normalization of bleeding times, reduced bone pain, elimination of hip fractures. Gaucher was the first disease in which enzyme-replacement therapy was used successfully. Recent studies have also led to an appreciation of the relationship between the carrier state for Gaucher’s disease and Parkinson’s disease (Aharon-Peretz et al. 2005).

A third example relates to oncogenetics and in particular genetic testing for breast and ovarian cancer (Robson and Offit 2007). Clustering of some forms of cancer in families has been well documented (Goldgar et al. 1994; Simchoni et al. 2006). Some women come from high-risk families with mothers and sisters, aunts, and cousins affected with breast and/or ovarian cancer. Gene mapping studies in multiple extended families with early-onset breast and ovarian cancer led to the identification of the BRCA1 and BRCA2 genes as harboring germline breast and ovarian cancer risk mutations. Among Ashkenazi Jews, three mutations are common and make genetic testing simple and relatively inexpensive (Oddoux et al. 1996). Genetic testing has led to prevention of cancers through life-preserving prophylactic oophorectomy and mastectomy. Ashkenazi Jews may also be in the vanguard for population-based genetic screening for breast and ovarian cancer risk. In 2009, Dr. Wendy Rubinstein and colleagues reported that almost half of Ashkenazi Jews identified with BRCA1/2 cancer-risk mutations have negative family histories for cancer. The absence of a family history confounds efforts toward presymptomatic carrier identification. Their model predicted that a genetic screening program would result in 2,811 fewer cases of ovarian cancer, with a life expectancy gain of 1.83 quality-adjusted life years among carriers at a cost of $8,300 (discounted) per year of quality-adjusted life gained (Rubinstein et al. 2009). Their recommendations were at odds with earlier recommendations of the US Preventive Services Task Force that population-based genetic screening for BRCA1/2 should not be offered.

## Toward a personalized medicine for the Jewish people based on genomics

Genome-wide analysis holds the prospect of understanding all of the genetic risks of the Jewish people and translating these into surveillance and treatment programs that will prevent disease or optimize therapy and minimize toxicity when disease does occur. Multiple genome-wide association studies (GWAS) for common diseases have been mounted for conditions that are either more prevalent among Ashkenazi Jews (Crohn disease, Parkinson disease) or premised to have less genetic heterogeneity compared with European or European–American populations (breast cancer, diabetes, bipolar disease, schizophrenia). Notably, these GWAS have identified disease-associated variants in the AJ population that were not identified in other populations (Avramopoulos et al. 2007; Liu et al. 2011; Nishioka et al. 2010; Shifman et al. 2008). Sequencing strategies hold the possibility of identifying both common and rare variants that affect disease risk as well as response to therapy and development of toxicity.

This work will be of importance in both the United States and Israel. Ashkenazi Jews are America’s largest genetic isolate, numbering some 6 million people or 2 % of the population. Based on patterns of participation in earlier genetic studies, it seems likely that members of this group will embrace new genomic medicine studies. All Jewish Diaspora groups are represented in Israel and receive their care through one of only four nationwide mandatory health care organizations. Linking their medical records (with adequate privacy safeguards) will provide an opportunity to identify the genetic factors that influence disease onset and progression and the therapeutic response.

## Electronic supplementary material

Below is the link to the electronic supplementary material.

Supplementary Table 1 Annotation of founder mutations in Jewish communities, based in part on information in the Israeli National Genetic Database http://www.goldenhelix.org/server/israeli/ and supporting literature and online databases. (XLS 51 kb)

## References

[CR1] Benjamin S (1983). The itinerary of Benjamin of Tudela: travels in the Middle Ages.

[CR2] Aharon-Peretz J, Badarny S, Rosenbaum H, Gershoni-Baruch R (2005). Mutations in the glucocerebrosidase gene and Parkinson disease: phenotype–genotype correlation. Neurology.

[CR3] Atzmon G, Hao L, Pe’er I, Velez C, Pearlman A, Palamara PF, Morrow B, Friedman E, Oddoux C, Burns E, Ostrer H (2010). Abraham’s children in the genome era: major Jewish diaspora populations comprise distinct genetic clusters with shared Middle Eastern Ancestry. Am J Hum Genet.

[CR4] Avramopoulos D, Lasseter VK, Fallin MD, Wolyniec PS, McGrath JA, Nestadt G, Valle D, Pulver AE (2007). Stage II follow-up on a linkage scan for bipolar disorder in the Ashkenazim provides suggestive evidence for chromosome 12p and the GRIN2B gene. Genet Med.

[CR5] Baron SW (1952). A social and religious history of the Jews.

[CR6] Behar DM, Garrigan D, Kaplan ME, Mobasher Z, Rosengarten D, Karafet TM, Quintana-Murci L, Ostrer H, Skorecki K, Hammer MF (2004). Contrasting patterns of Y chromosome variation in Ashkenazi Jewish and host non-Jewish European populations. Hum Genet.

[CR7] Behar DM, Metspalu E, Kivisild T, Rosset S, Tzur S, Hadid Y, Yudkovsky G, Rosengarten D, Pereira L, Amorim A, Kutuev I, Gurwitz D, Bonne-Tamir B, Villems R, Skorecki K (2008). Counting the founders: the matrilineal genetic ancestry of the Jewish Diaspora. PLoS ONE.

[CR8] Behar DM, Yunusbayev B, Metspalu M, Metspalu E, Rosset S, Parik J, Rootsi S, Chaubey G, Kutuev I, Yudkovsky G, Khusnutdinova EK, Balanovsky O, Semino O, Pereira L, Comas D, Gurwitz D, Bonne-Tamir B, Parfitt T, Hammer MF, Skorecki K, Villems R (2010). The genome-wide structure of the Jewish people. Nature.

[CR9] Ben-Sasson HH (1976). A history of the Jewish people.

[CR10] Biran A, Naveh J (1993). An aramaic stele fragment from Tel Dan. Israel Explor J.

[CR11] Bonne-Tamir B, Ashbel S, Modai J (1977). Genetic markers in Libyan Jews. Hum Genet.

[CR12] Bonne-Tamir B, Ashbel S, Bar-Shani S (1978). Ethnic communities in Israel: the genetic blood markers of the Babylonian Jews. Am J Phys Anthropol.

[CR13] Bonne-Tamir B, Ashbel S, Bar-Shani S (1978). Ethnic communities in Israel: the genetic blood markers of the Moroccan Jews. Am J Phys Anthropol.

[CR14] Bray SM, Mulle JG, Dodd AF, Pulver AE, Wooding S, Warren ST (2010). Signatures of founder effects, admixture, and selection in the Ashkenazi Jewish population. Proc Natl Acad Sci USA.

[CR15] Bryc K, Velez C, Karafet T, Moreno-Estrada A, Reynolds A, Auton A, Hammer M, Bustamante CD, Ostrer H (2010). Colloquium paper: genome-wide patterns of population structure and admixture among Hispanic/Latino populations. Proc Natl Acad Sci USA.

[CR16] Campbell CL, Palamara PF, Dubrovsky M, Botigué LR, Fellous M, Atzmon G, Oddoux C, Pearlman A, Hao L, Henn BM, Burns E, Bustamante CD, Comas D, Friedman E, Pe’er I, Ostrer H (2012) North African Jewish and non-Jewish populations form distinctive, orthogonal clusters. Proc Natl Acad Sci USA (in press) 10.1073/pnas.1204840109 PMC3427049 22869716

[CR17] Carmelli D, Cavalli-Sforza LL (1979). The genetic origin of the Jews: a multivariate approach. Hum Biol.

[CR18] Charrow J (2009). Enzyme replacement therapy for Gaucher disease. Expert Opin Biol Ther.

[CR19] Chi J, Heath S, New York University, Institute for the Study of the Ancient World (2011). Edge of empires: pagans, Jews, and Christians at Roman Dura-Europos.

[CR20] Cohen SJD (1999). The beginnings of Jewishness.

[CR21] Einstein A, Beck A, Havas P (1987). The collected papers of Albert Einstein.

[CR22] Fishberg M (1911). The Jews: a study of race and environment.

[CR23] Goldgar DE, Easton DF, Cannon-Albright LA, Skolnick MH (1994). Systematic population-based assessment of cancer risk in first-degree relatives of cancer probands. J Natl cancer Inst.

[CR24] Goldstein DB (2008). Jacob’s legacy: a genetic view of Jewish history.

[CR25] Goldstein P, Evans H (2012). A convenient hatred: the history of antisemitism.

[CR26] Gonen R (2002). The quest for the ten lost tribes of Israel: to the ends of the earth.

[CR27] Goodman RM (1974). Various genetic traits and diseases among the Jewish ethnic groups. Birth Defects Orig Artic Ser.

[CR28] Goodman Genetic (1979). Disorders among the Jewish people.

[CR29] Hammer MF, Redd AJ, Wood ET, Bonner MR, Jarjanazi H, Karafet T, Santachiara-Benerecetti S, Oppenheim A, Jobling MA, Jenkins T, Ostrer H, Bonne-Tamir B (2000). Jewish and Middle Eastern non-Jewish populations share a common pool of Y-chromosome biallelic haplotypes. Proc Natl Acad Sci USA.

[CR30] Hordes SM (2005). To the end of the earth: a history of the crypto-Jews of New Mexico.

[CR31] Isaac BH (1998). The near east under Roman rule: selected papers.

[CR32] Jacobs J (1899). Are Jews Jews?. Pop Sci Mon.

[CR33] Kaback MM (2001). Screening and prevention in Tay-Sachs disease: origins, update, and impact. Adv Genet.

[CR34] Karlin S, Kenett R, Bonne-Tamir B (1979). Analysis of biochemical genetic data on Jewish populations II. Results and interpretations of heterogeneity indices and distance measures with respect to standards. Am J Hum Genet.

[CR35] Kobyliansky E, Micle S, Goldschmidt-Nathan M, Arensburg B, Nathan H (1982). Jewish populations of the world: genetic likeness and differences. Ann Hum Biol.

[CR36] Kopelman NM, Stone L, Wang C, Gefel D, Feldman MW, Hillel J, Rosenberg NA (2009). Genomic microsatellites identify shared Jewish ancestry intermediate between Middle Eastern and European populations. BMC Genet.

[CR37] Listman JB, Hasin D, Kranzler HR, Malison RT, Mutirangura A, Sughondhabirom A, Aharonovich E, Spivak B, Gelernter J (2010). Identification of population substructure among Jews using STR markers and dependence on reference populations included. BMC Genet.

[CR38] Liu X, Cheng R, Verbitsky M, Kisselev S, Browne A, Mejia-Sanatana H, Louis ED, Cote LJ, Andrews H, Waters C, Ford B, Frucht S, Fahn S, Marder K, Clark LN, Lee JH (2011). Genome-wide association study identifies candidate genes for Parkinson’s disease in an Ashkenazi Jewish population. BMC Med Genet.

[CR39] Livshits G, Sokal RR, Kobyliansky E (1991). Genetic affinities of Jewish populations. Am J Hum Genet.

[CR40] McKusick VA (1973). Ethnic distribution of disease in non-Jews. Israel J Med Sci.

[CR41] Motulsky AG, Vandepitte J, Fraser GR (1966). Population genetic studies in the Congo I. Glucose-6-phosphate dehydrogenase deficiency, hemoglobin S, and malaria. Am J Hum Genet.

[CR42] Nebel A, Filon D, Brinkmann B, Majumder PP, Faerman M, Oppenheim A (2001). The Y chromosome pool of Jews as part of the genetic landscape of the Middle East. Am J Hum Genet.

[CR43] Nebel A, Filon D, Faerman M, Soodyall H, Oppenheim A (2005). Y chromosome evidence for a founder effect in Ashkenazi Jews. Eur J Hum Genet.

[CR44] Nishioka K, Vilarino-Guell C, Cobb SA, Kachergus JM, Ross OA, Wider C, Gibson RA, Hentati F, Farrer MJ (2010). Glucocerebrosidase mutations are not a common risk factor for Parkinson disease in North Africa. Neurosci Lett.

[CR45] Oddoux C, Struewing JP, Clayton CM, Neuhausen S, Brody LC, Kaback M, Haas B, Norton L, Borgen P, Jhanwar S, Goldgar D, Ostrer H, Offit K (1996). The carrier frequency of the BRCA2 6174delT mutation among Ashkenazi Jewish individuals is approximately 1 %. Nat Genet.

[CR46] Ostrer H (2001). A genetic profile of contemporary Jewish populations. Nat Rev Genet.

[CR47] Ostrer H (2011). Changing the game with whole exome sequencing. Clin Genet.

[CR48] Parfitt T (2002). The lost tribes of Israel: the history of a myth.

[CR49] Risch N, Tang H, Katzenstein H, Ekstein J (2003). Geographic distribution of disease mutations in the Ashkenazi Jewish population supports genetic drift over selection. Am J Hum Genet.

[CR50] Robson M, Offit K (2007). Clinical practice. Management of an inherited predisposition to breast cancer. N Engl J Med.

[CR51] Rosenberg NA, Woolf E, Pritchard JK, Schaap T, Gefel D, Shpirer I, Lavi U, Bonne-Tamir B, Hillel J, Feldman MW (2001). Distinctive genetic signatures in the Libyan Jews. Proc Natl Acad Sci USA.

[CR52] Rubinstein WS, Jiang H, Dellefave L, Rademaker AW (2009). Cost-effectiveness of population-based BRCA1/2 testing and ovarian cancer prevention for Ashkenazi Jews: a call for dialogue. Genet Med.

[CR53] Semino O, Passarino G, Oefner PJ, Lin AA, Arbuzova S, Beckman LE, De Benedictis G, Francalacci P, Kouvatsi A, Limborska S, Marcikiae M, Mika A, Mika B, Primorac D, Santachiara-Benerecetti AS, Cavalli-Sforza LL, Underhill PA (2000). The genetic legacy of Paleolithic *Homo sapiens* sapiens in extant Europeans: a Y chromosome perspective. Science.

[CR54] Shanks H (1988). Ancient Israel: a short history from Abraham to the Roman destruction of the temple.

[CR55] Shifman S, Johannesson M, Bronstein M, Chen SX, Collier DA, Craddock NJ, Kendler KS, Li T, O’Donovan M, O’Neill FA, Owen MJ, Walsh D, Weinberger DR, Sun C, Flint J, Darvasi A (2008). Genome-wide association identifies a common variant in the reelin gene that increases the risk of schizophrenia only in women. PLoS Genet.

[CR56] Simchoni S, Friedman E, Kaufman B, Gershoni-Baruch R, Orr-Urtreger A, Kedar-Barnes I, Shiri-Sverdlov R, Dagan E, Tsabari S, Shohat M, Catane R, King MC, Lahad A, Levy-Lahad E (2006). Familial clustering of site-specific cancer risks associated with BRCA1 and BRCA2 mutations in the Ashkenazi Jewish population. Proc Natl Acad Sci USA.

[CR57] Slatkin M (2004). A population-genetic test of founder effects and implications for Ashkenazi Jewish diseases. Am J Hum Genet.

[CR58] Stillman N (1991). The Jews of Arab lands.

[CR59] van Baal S, Zlotogora J, Lagoumintzis G, Gkantouna V, Tzimas I, Poulas K, Tsakalidis A, Romeo G, Patrinos GP (2010). ETHNOS : a versatile electronic tool for the development and curation of national genetic databases. Hum Genomics.

[CR60] Velez C, Palamara PF, Guevara-Aguirre J, Hao L, Karafet T, Guevara-Aguirre M, Pearlman A, Oddoux C, Hammer M, Burns E, Pe’er I, Atzmon G, Ostrer H (2011). The impact of Converso Jews on the genomes of modern Latin Americans. Hum Genet.

[CR61] Wistrich RS (2010). A lethal obsession: anti-Semitism from antiquity to the global Jihad.

[CR62] Zalloua PA, Xue Y, Khalife J, Makhoul N, Debiane L, Platt DE, Royyuru AK, Herrera RJ, Hernanz DF, Blue-Smith J, Wells RS, Comas D, Bertranpetit J, Tyler-Smith C (2008). Y-chromosomal diversity in Lebanon is structured by recent historical events. Am J Hum Genet.

